# Extended real-world experience with the ILUVIEN® (fluocinolone acetonide) implant in the United Kingdom: 3-year results from the Medisoft® audit study

**DOI:** 10.1038/s41433-021-01542-w

**Published:** 2021-05-10

**Authors:** Clare Bailey, Usha Chakravarthy, Andrew Lotery, Geeta Menon, James Talks, for the Medisoft Audit Group

**Affiliations:** 1 Department of Ophthalmology, Bristol Eye Hospital, Bristol, UK; 2 Belfast Health and Social Care Trust, Belfast, UK; 3 University Hospital Southampton, Southampton, UK; 4 Frimley Park Hospital, Frimley, UK; 5 Newcastle Upon Tyne Hospitals, Newcastle Upon Tyne, UK

**Keywords:** Macular degeneration, Glaucoma, Retinal diseases

## Abstract

**Background:**

This study aimed to assess the long-term effectiveness of the 0.2 μg/day fluocinolone acetonide (FAc) implant over ≥3 years for patients with diabetic macular oedema.

**Methods:**

A retrospective audit of pseudo-anonymised data from patients with chronic diabetic macular oedema (cDMO) and treated with the FAc implant across 14 UK clinical sites. Safety and clinical effectiveness were measured.

**Results:**

Two-hundred and fifty-six eyes had ≥3 years of follow-up (mean 4.28 years), during which a mean of 1.14 FAc implants were used per eye. Mean best-recorded visual acuity (BRVA) increased from 52.6 to 56.7 letters at month 3 and remained stable thereafter; this trend was also seen in pseudophakic eyes. The proportion of patients attaining a BRVA ≥6/12 increased from 17% at baseline to 27% 1 month after FAc implant and remained stable above 30% from month 12 onwards. Eyes with no prior history of intraocular pressure (IOP)-related events required significantly less treatment-emergent IOP-lowering medication than those with a prior history of IOP events (17.9% vs. 50.0% of eyes; *p* < 0.001). The incidence of an IOP increase of ≥10 mmHg, use of IOP-lowering medication, laser trabeculoplasty and IOP-lowering surgery was 28.9%, 29.7%, 0.8% and 2.7%, respectively, for the whole cohort. There were significant reductions in mean central foveal thickness and macular volume (*p* < 0.001).

**Conclusions:**

The FAc implant was well tolerated, with predictable and manageable IOP-related events while delivering a continuous microdose of corticosteroid to eyes with cDMO, providing prolonged vision preservation and a reduced number of treatments.

## Introduction

The goal of treating diabetic macular oedema (DMO) is to preserve or improve vision by reducing macular swelling [1]. Anti-vascular endothelial growth factor (VEGF) treatment is the first-line treatment for centre-involving DMO [2–4]. However, up to 66% of patients can have an insufficient response, despite initial intensive monthly anti-VEGF therapy [5]. This presents a substantial treatment burden for patients and healthcare providers; patients may be reluctant to receive such treatment and may miss clinic follow-up visits because of other hospital appointments [6]. The current Covid-19 pandemic has shown that it would be beneficial to have treatments that require fewer injection visits and/or less frequent clinic visits.

DMO can recur once first-line treatment with intravitreal anti-VEGF therapies is discontinued or if the interval between treatments is lengthened [7]. It is known that patients who receive fewer injections of anti-VEGF treatment for DMO achieve worse visual gains than patients who are intensively treated in randomised clinical trials, possibly because frequent, routine injections are harder to maintain in clinical practice than in clinical trials [8, 9].

Intravitreal corticosteroid implants can be used in patients with DMO who have not had a sufficient response to prior therapy [4, 10]. There are two approved therapies: the dexamethasone implant and the fluocinolone acetonide (FAc) implant [11, 12], the latter of which (ILUVIEN®, Alimera Sciences Limited, Aldershot, UK) provides a sustained, low-dose release of 0.2 μg of FAc per day for up to 36 months [13].

We previously reported the interim results for an initial group of patients in a real-world evaluation of 0.2 μg/day FAc implant. The data were derived from an electronic medical record (EMR) (Medisoft®, Leeds, UK), for 2 years of follow-up post-injection of the FAc implant [14]. Results showed that the implant had a favourable safety profile, with improvements in visual acuity (VA) and retinal morphology [14]. This provided further evidence of the value of the FAc implant for treating patients with persistent or recurrent DMO despite treatment but did not provide evidence on its long-term use in the whole cohort.

The present study is a further evaluation of data from patients in this database, evaluating effectiveness and safety outcomes at ≥3 years from 14 clinical sites in the UK. This report describes one of the largest cohorts with chronic DMO to date, with data evaluating long-term, real-world use of the 0.2 μg/day FAc implant [15, 16]. This is important for identifying suitable patients and elucidating the clinical outcomes of this therapy.

## Materials and methods

This was a retrospective audit of data for patients who had received the FAc implant for the licensed indication of chronic DMO at any of the 14 participating centres in the UK.

This analysis was conducted on data extracted in October 2019 in accordance with the Declaration of Helsinki and the UK’s Data Protection Act; the previous published analysis was conducted on data extracted in August 2016 [14]. Caldicott guardian approval was obtained from each site. Processes for data automation and extraction have been described previously [14]. Data available in the extracted set included: baseline clinical and disease characteristics; prior treatments for DMO; intraocular pressure (IOP) when recorded and treatments administered if an increase in IOP was noted; any additional ocular treatments administered for DMO after FAc implant; VA; and central subfield foveal thickness if measured and entered into the electronic record.

Change from baseline for the mean and median best-recorded visual acuity (BRVA) was calculated and assessed for each group, stratified by baseline VA over 48 months. The central foveal thickness and macular volume were analysed from available baseline values and follow-up data at the first and last visits following injection of the 0.2 μg/day FAc.

### Data and statistical analysis

Data are reported either as mean  ±  standard deviation (SD) or as a percentage of eyes or patients, unless otherwise stated. All *p* values were calculated based on a Pearson chi-square test for the difference between eyes with and without a prior history of IOP-related events for IOP-related outcomes.

## Results

### Study population

Data were available for 256 eyes (227 patients) with a minimum of 3 years of follow-up (mean follow-up duration of 4.28 years). Demographics, baseline characteristics and prior treatments are reported in Supplementary Table S2. The majority of eyes were pseudophakic (88.7%). After 36 months following the initial FAc implant, IOP data were available for 124 eyes, and VA data were available for 162 eyes. After 48 months, IOP data were available for 84 eyes, and VA data were available for 120 eyes. The study was designed to collect all available VA and IOP values for all patients in this cohort from baseline to 36 months. This approach aimed to maximise the number of patients for which 3 years of safety data (IOP and cataract-related events) were collected, as safety outcomes were the main focus of this study. All eyes had safety data captured for 3 years.

The mean duration of DMO was 4.4 ± 2.9 years. The majority of treated eyes had been recorded as having received prior therapy for DMO (92.6%) before receiving the 0.2 μg/day FAc implant, with most having intravitreal anti-VEGF last before FAc implant (69.1%; Supplementary Table S3).

### Number of FAc implants

Overall, a mean of 1.14 FAc implants were used per eye (293 injections in 256 eyes) over the entire course of follow-up. The mean time to the injection of the second implant was 1160.7 days (~3.2 years; range 357–1842 days). No patient received more than two FAc implants during the period of follow-up.

### Visual outcomes

**Fig. 1:**
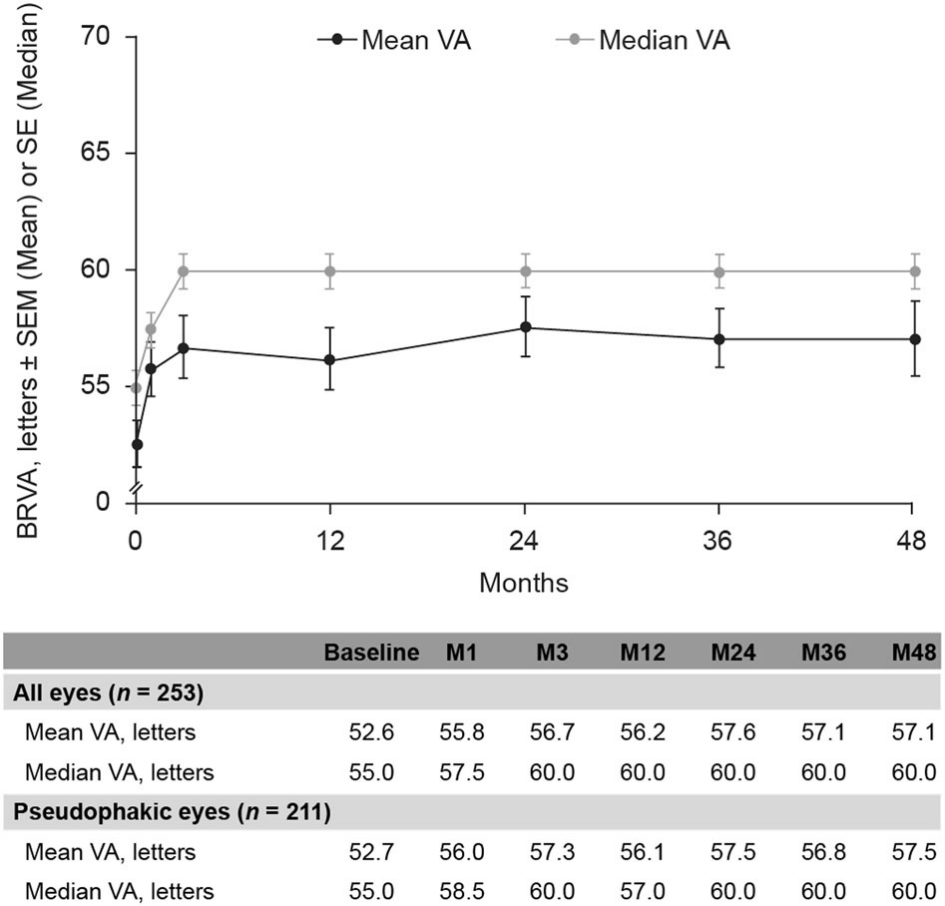
BRVA for all eyes (shown in both the figure and the table) and pseudophakic eyes (shown in the table only) over 48 months. Error bars represent SEM (Mean) or SE (Median). BRVA best-recorded visual acuity, SE standard error, SEM standard error of the mean, VA visual acuity.

Mean baseline BRVA was 52.6 letters (*n* = 253). This increased to 56.7 letters at month 3 (*n* = 144) and remained stable for the follow-up period of ≥3 years, with a similar trend for eyes that were pseudophakic at baseline (Fig. 1).

**Fig. 2:**
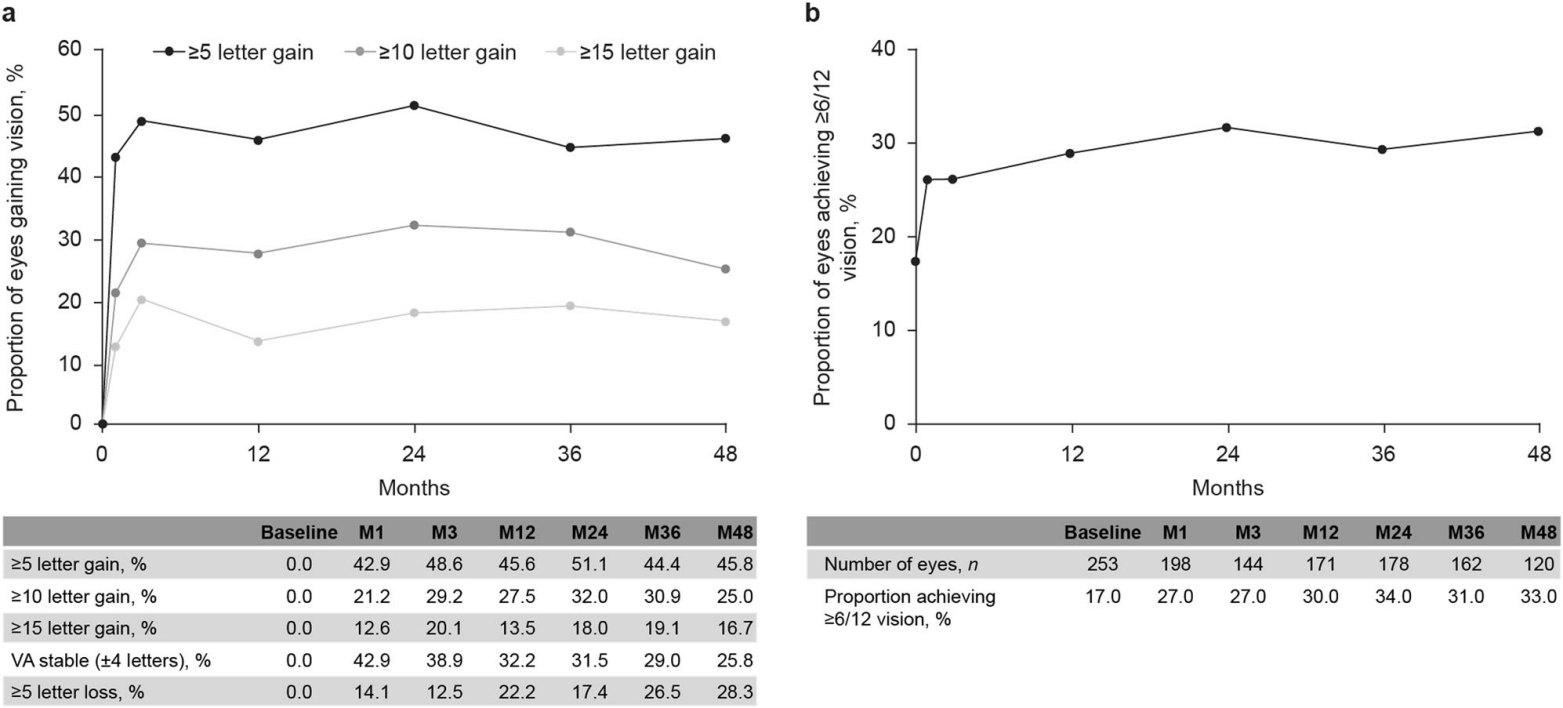
BRVA outcomes for all eyes over 48 months. Proportion of eyes (**a**) gaining 1, 2 or 3 lines of improvement in BRVA and (**b**) achieving ≥6/12 vision BRVA best-recorded visual acuity, VA visual acuity.

The overall percentage of eyes with stable vision or improvement (defined as any gain, or any loss equal or less than 4 letters from baseline) was 73% at month 36 and 72% at month 48. The proportion of eyes gaining ≥5, ≥10 and ≥15 letters is shown in Fig. 2a, and the proportion of patients achieving ≥6/12 vision is shown in Fig. 2b.

### Management of IOP and the impact of prior IOP-related events

The baseline mean IOP was 15.8 mmHg, and the mean IOP remained in the normal range (<21 mmHg) for those with long-term follow-up (Supplementary Table S4).

**Fig. 3:**
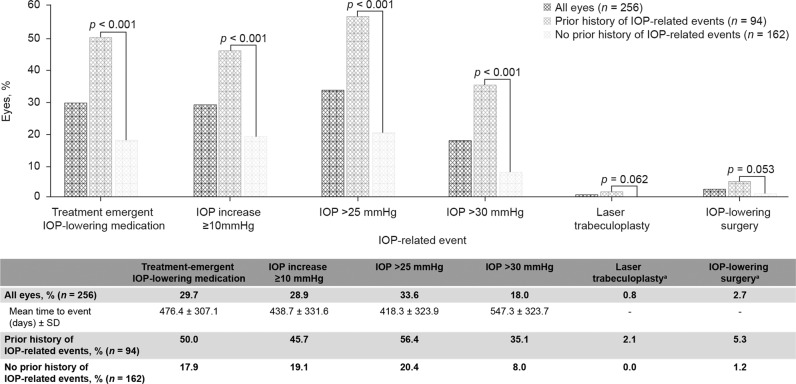
Impact of prior IOP-related events. ^a^Time-to-event analyses were not performed for laser trabeculoplasty or IOP-lowering surgery, as the number of events was very small, and the data could have been significantly skewed by outliers. IOP intraocular pressure, SD standard deviation.

Prior to receiving the 0.2 μg/day FAc implant, 16.0% of eyes had already received IOP-lowering medication; 3.9% of eyes had experienced IOP >30 mmHg before baseline, and 0.4% of eyes had required laser trabeculoplasty. The incidences of IOP-related events over the 36 months following FAc implant treatment, along with the mean time to the event, are shown in Fig. 3. At baseline, 94 eyes (36.7%) had a history of IOP-related events prior to 0.2 μg/day FAc implant injection. There was a significant difference in both the incidence of treatment-emergent IOP-lowering medication and in the incidence of IOP >30 mmHg in eyes with and without a prior history of IOP-related events (*p* < 0.001; Fig. 3).

### Retinal structural changes

In the subset of patients in whom optical coherence tomography thickness measures were recorded (*n* = 66), the mean central foveal thickness decreased by 20% from baseline to the first visit post-FAc implant injection (from 460.3 to 368.5 μm) and by 26% from baseline to the last visit post-FAc implant injection (to 340.5 μm). Both of these reductions were significant (*p* < 0.001). Macular volume decreased from 9.9 mm^3^ at baseline to 9.2 mm^3^ at the first visit (7% reduction; *p* = 0.028) and to 8.8 mm^3^ at the last visit (11% reduction; *p* < 0.001).

### Treatments used after 0.2 μg/day FAc implant

The majority of patients received macular laser and/or intravitreal treatments prior to FAc implant injection; 55.9% of eyes over the 36 months following implant injection received additional treatment. The time periods in which each type of additional treatment was added are shown in Supplementary Table S5.

## Discussion

The current study used structured EMR data from 14 UK retina centres in the UK to assess real-life outcomes following the use of 0.2 μg/day FAc implant for the treatment of chronic DMO. On average, the majority of treated eyes had moderate visual impairment and had previously been treated with intravitreal therapy, including anti-VEGFs and other corticosteroids, before treatment with the FAc implant. Our analysis demonstrates that ILUVIEN led to the maintenance or improvement (by a median of five letters) in the majority of patients for ≥3 years while also improving retinal morphology and having an overall favourable safety profile.

The current results were obtained in a real-life setting where treatment was initiated in eyes with VA ranging from 5 to 85 Early Treatment Diabetic Retinopathy Study letters who had previously been treated extensively before being treated with the FAc implant. VA outcomes were in line with the previous findings reported by Bailey et al. in 2017 and consistent with other real-world studies [14, 15].

Analyses of VA outcomes showed that the proportion of patients with stable VA or achieving a VA of ≥6/12 was comparable with results from the Fluocinolone Acetonide for Diabetic Macular Edema (FAME) study over 36 months. The current analysis also provides additional insight, as it showed there was a doubling of the proportion of patients achieving a VA of ≥6/12 by month 24 and that this effect was still evident after 4 years. These findings are important considerations, as they are expected to benefit a patient’s quality of life through maintenance of existing functional vision or re-enable a patient to drive (in the UK, the legal minimum requirement for driving is binocular VA of 6/12 [17]).

It is notable that the proportion of patients with an improvement in BRVA of ≥15 letters over 36 months in the FAME study (34.0%) was greater than in this present study [13]. It is important to note that the current study includes patients with high VA at baseline, and they will experience a ceiling effect and be unable to gain 15 letters. Furthermore, the current study reports effects up to 4 years in patients where data were collected and that patients were monitored in real clinical practices in the UK and had been treated extensively prior to intravitreal injection of the FAc implant. Regardless of this, the Medisoft dataset demonstrates consistency of effectiveness in one of the largest real-world datasets currently available for this therapy [15, 18–21].

Manageable and predictable levels of IOP change were observed during the current study with mean IOP remaining below 21 mmHg throughout the study period. The additional analyses based on prior IOP treatment history should also be of value to physicians, as these suggest that the occurrence of an IOP event may be a good predictor of future events. Indeed, eyes with no prior history of IOP-related events required significantly less treatment-emergent IOP-lowering medication than those with a prior history of IOP. This trend was also reflected in the other IOP-related events groups and is consistent with previous analyses [14]. A new aspect in the current analysis was the calculation of mean time to IOP-related events, which showed that events occurred on average during the second year of FAc therapy, further reinforcing the importance of quarterly IOP monitoring following FAc treatment.

There was a large reduction in the overall use of intravitreal treatments following FAc implant injection. The time at which these treatments were introduced was evenly distributed across the first, second and third year. It should be noted that it is likely some centres did not administer adjunctive treatment based on the local interpretation of NICE guidance. For some eyes, supplemental therapy involved a second FAc implant (mean number of 1.14 implants). In these cases, the mean time to the second implant was around 3.2 years and shows that the FAc implant can provide 3 years (or more) of therapy and reinforces its low clinical and treatment burden in real-world UK practice. The number of second injections is lower than that reported in the FAME study (1.3 injections over 3 years) [22]. This may be particularly relevant in the current pandemic, in which it is beneficial to have treatments available that may facilitate less frequent clinic visits and fewer injection visits.

Potential limitations of this study include the confounding effects of supplementary treatments; however, this reflects the real-world practice for managing chronic DMO, where the use of supplementary treatments such as laser photocoagulation is commonplace, even with first-line anti-VEGF agents. Furthermore, these supplementary treatments might be addressing the continued neovascularisation in the patients with baseline proliferative diabetic retinopathy rather than persistent oedema. The retrospective nature of this study meant that missing data points could not be obtained, even though the data were originally entered prospectively. Furthermore, as this was an open-label study, there was no validation of the disease state, and the quality of the data depended on the accurate completion of the electronic records. Patients’ correction of VA at each visit in real-world studies may lead to an underestimate of the real vision changes, although this will also more accurately predict the visual outcomes that patients should experience themselves.

In conclusion, this is one of the largest and longest studies to date demonstrating the long-term effectiveness and safety of the FAc implant for the treatment of chronic DMO in routine clinical practice for ≥3 years. Patients with good vision at baseline and who received the implant maintained their good vision throughout ≥3 years, implying that the implant satisfies the DMO treatment goal of achieving or improving vision as a long-term outcome. The mean time to second implant and the reduction in supplementary treatments following injection show that the implant also achieves the goal of reducing visits to the clinic and the number of injections. This study also confirms the predictable and manageable side-effect profile of the FAc implant that is more favourable in the absence of prior IOP-related events.

## Summary

### What was known before

- Anti-VEGF treatment is approved for treatment in DMO; however, up to 66% of patients can have an insufficient response.
- The 0.2 μg/day FAc implant is indicated for the treatment of chronic DMO in patients insufficiently responsive to prior therapies.
- Previous 2-year follow-up of real-world FAc implant use showed comparable efficacy and safety outcomes to the pivotal FAME trial.

### What this study adds

- This is the first demonstration of a single injection of the FAc implant providing stable or improved VA in the majority of patients (equal or greater than 72%) for ≥3 years, with sustained benefits in patients with good baseline VA. Along with the substantial reduction in treatment burden over ≥3 years, this suggests that the FAc implant achieves the long-term goals of treating persistent or recurring DMO: achieving or improving vision and reducing treatment burden.
- The mean IOP was stable and remained below 21 mmHg throughout ≥3 years of treatment. Patients with a prior history of IOP-related adverse events were much more likely to require treatment-emergent IOP-lowering medication; however, the mean time to this IOP event was in the second year following FAc implant.

## Supplementary information

Supplementary Table S1

Supplementary Table S2

Supplementary Table S3

Supplementary Table S4

Supplementary Table S5
