# Supplementary material for: Extended real-world experience with the ILUVIEN® (fluocinolone acetonide) implant in the United Kingdom: 3-year results from the Medisoft® audit study
Source: Eye (Lond). 2021 May 10;36(5):1012–8. doi: 10.1038/s41433-021-01542-w (PMC8107780; doi:10.1038/s41433-021-01542-w)
Supplement: Supplementary file 1 — Supplementary Table S1 [file 41433_2021_1542_MOESM1_ESM.docx]

**Supplementary Table S1** Medisoft Audit Group contributors

| Name | Affiliation |
| --- | --- |
| Miss Clare Bailey (Principal Investigator) | Bristol Eye Hospital |
| Mr Ahmed Kamal | Aintree University Hospitals |
| Professor Usha Chakravarthy | Belfast Health and Social Care Trust |
| Professor Faruque Ghanchi | Bradford Teaching Hospitals |
| Mr Karnesh Patel | Calderdale Royal Hospital |
| Professor Geeta Menon | Frimley Park Hospital |
| Ms Emily Fletcher | Gloucestershire Hospitals |
| Mr Martin McKibbin | Leeds Teaching Hospitals |
| Mr Narendra Dhingra | Mid Yorkshire Hospitals |
| Ms Bushra Mustaq | Birmingham Midland Eye Centre |
| Mr Christopher Brand | Sheffield Teaching Hospitals |
| Mr James Talks | Newcastle upon Tyne Hospitals |
| Professor Andrew Lotery | University Hospital Southampton |
| Miss Helen Palmer | Queen Elizabeth Hospital Birmingham |
