# Supplementary material for: Extended real-world experience with the ILUVIEN® (fluocinolone acetonide) implant in the United Kingdom: 3-year results from the Medisoft® audit study
Source: Eye (Lond). 2021 May 10;36(5):1012–8. doi: 10.1038/s41433-021-01542-w (PMC8107780; doi:10.1038/s41433-021-01542-w)
Supplement: Supplementary file 2 — Supplementary Table S2 [file 41433_2021_1542_MOESM2_ESM.docx]

**Supplementary Table S2** Patient demographics and baseline characteristics

| Characteristic | Patients (*n* = 227; 256 DMO eyes) |
| --- | --- |
| Gender | 51.1% male / 48.9% female |
| Mean age | 66.9 ± 10.6 years |
| Age at time of diabetes diagnosis | 46.2 ± 16.9 years |
| Type of diabetes, % |  |
| Type I | 17.2% |
| Type II | 73.6% |
| Other/missing | 9.2% |
| Time since first episode of DMO | 4.4 ± 2.9 years |
| Time since first intravitreal therapy | 1.4 ± 1.4 years |
| Time from last therapy received for DMO | 0.55 ± 0.73 years |
| Lens status | 88.7% pseudophakic (227) : 11.3% (29) phakic |
| Combined cataract surgery/0.2 μg/day FAc implant (same day) | 6.6% (17/256 eyes) |
| Mean time to cataract surgery for those not having surgery on the day of implant | 497.7 ± 488.3 days |
| Mean duration of follow-up | 4.3 years (1562 days; SD 247.2 days; range 1100–2068) |
| Proliferative diabetic retinopathy | 58.9% (109/185 eyes) |
| Mild/moderate | 56.2% (104/185 eyes) |
| Any prior macular laser or intravitreal treatment | 92.6% (237/256 eyes) |
| Any prior macular laser treatment | 31.6% (81/256 eyes) |
| Prior panretinal photocoagulation laser | 24.6% (63/256 eyes) |
| Any prior intravitreal treatment | 84.8% (217/256 eyes) |
| Intravitreal corticosteroid | 32.0% (82/256 eyes) |
| Intravitreal anti-VEGF | 79.7% (204/256 eyes) |

*DMO* diabetic macular oedema, *FAc* fluocinolone acetonide, *SD* standard deviation, *VEGF* vascular endothelial growth factor.
