# Supplementary material for: Extended real-world experience with the ILUVIEN® (fluocinolone acetonide) implant in the United Kingdom: 3-year results from the Medisoft® audit study
Source: Eye (Lond). 2021 May 10;36(5):1012–8. doi: 10.1038/s41433-021-01542-w (PMC8107780; doi:10.1038/s41433-021-01542-w)
Supplement: Supplementary file 3 — Supplementary Table S3 [file 41433_2021_1542_MOESM3_ESM.docx]

**Supplementary Table S3** Last treatment prior to receiving 0.2 μg/day FAc implant

|  | Percentage of eyes | Mean time between last treatment and 0.2 μg/day FAc implant (years) |
| --- | --- | --- |
| None | 7.4% (19/256 eyes) |  |
| Laser photocoagulation | 10.9% (28/256 eyes) | 1.24 ± 1.38 |
| Intravitreal steroid | 12.5% (32/256 eyes) | 0.72 ± 0.89 |
| Intravitreal anti‑VEGF | 69.1% (177/256 eyes) | 0.41 ± 0.44 |

*FAc* fluocinolone acetonide, *VEGF* vascular endothelial growth factor.
