# Supplementary material for: Extended real-world experience with the ILUVIEN® (fluocinolone acetonide) implant in the United Kingdom: 3-year results from the Medisoft® audit study
Source: Eye (Lond). 2021 May 10;36(5):1012–8. doi: 10.1038/s41433-021-01542-w (PMC8107780; doi:10.1038/s41433-021-01542-w)
Supplement: Supplementary file 4 — Supplementary Table S4 [file 41433_2021_1542_MOESM4_ESM.docx]

**Supplementary Table S4** Mean IOP and mean change in IOP from baseline

|  | Baseline | M1 | M3 | M12 | M24 | M36 | M48 |
| --- | --- | --- | --- | --- | --- | --- | --- |
| Mean IOP, mmHg | 15.8 | 17.3 | 18.4 | 18.9 | 18.8 | 18.2 | 18.0 |
| Mean change in IOP, mmHg | 0.0 | 1.6 | 2.5 | 2.8 | 2.9 | 2.2 | 1.9 |

*IOP* intraocular pressure.
