# Supplementary material for: Extended real-world experience with the ILUVIEN® (fluocinolone acetonide) implant in the United Kingdom: 3-year results from the Medisoft® audit study
Source: Eye (Lond). 2021 May 10;36(5):1012–8. doi: 10.1038/s41433-021-01542-w (PMC8107780; doi:10.1038/s41433-021-01542-w)
Supplement: Supplementary file 5 — Supplementary Table S5 [file 41433_2021_1542_MOESM5_ESM.docx]

**Supplementary Table S5** Use of supplementary treatments over 36 months

|  | Percentage of eyes; mean number of treatments | | | | |
| --- | --- | --- | --- | --- | --- |
|  | Pre-implant^a^ | 36  months | 0–12  months | 12–24  months | 24–36  months |
| Any macular laser or intravitreal treatment | 92.6% | 55.9% | 34.0% | 40.6% | 35.2% |
|  | – | 7.3 | 3.3 | 4.0 | 3.7 |
| Macular laser photocoagulation | 31.6% | 10.5% | 5.5% | 4.7% | 3.9% |
|  | – | 1.4 | 1.1 | 1.1 | 1.0 |
| Intravitreal treatment | 84.8% | 52.0% | 32.0% | 37.5% | 33.2% |
|  | – | 7.5 | 3.3 | 4.2 | 3.8 |
| Intravitreal steroid | 32.0% | 9.4% | 3.5% | 3.1% | 5.5% |
|  | – | 1.5 | 1.0 | 1.1 | 1.3 |
| Intravitreal anti‑VEGF | 79.7% | 48.8% | 28.9% | 36.3% | 31.3% |
|  | – | 7.7 | 3.5 | 4.3 | 3.8 |

^a^Percentage of eyes receiving treatment prior to the fluocinolone acetonide implant.

*VEGF* vascular endothelial growth factor
